# Incidence and Outcomes of Brucella Endocarditis in a High-Prevalence Area: A Single-Center Study

**DOI:** 10.1007/s44197-024-00232-6

**Published:** 2024-06-03

**Authors:** Shufang Pan, Yunyue Zhao, Kaixiang Zhou, Shuru Chen, Miriban Maimaitiming, Jing Wu, Maimaitiaili Tuerxun, Yutian Chong, Jianyun Zhu

**Affiliations:** 1 Department of Infectious Diseases, The Third Affiliated Hospital of Sun Yat-sen University, Guangzhou, 510630 China; 2 Department of Infectious Diseases, The First People’s Hospital of Kashi Prefecture, Kashi, 844000 China; 3 Department of Rheumatology, The Third Affiliated Hospital of Sun Yat-sen University, Zhao Qing Hospital, Zhaoqing, 526000 China; 4 Department of Cardiology, The Third Hospital of Sun Yat-sen University, Guangzhou, 510630, China

**Keywords:** Brucella endocarditis, Bacterial endocarditis, Brucellosis, Bacterial zoonoses

## Abstract

**Objective:**

To analyze the clinical characteristics of Brucella endocarditis (BE) and observe the factors related to death to provide guidance for clinical treatment.

**Methods:**

This study examined all patients with BE admitted to The First People’s Hospital of Kashi Prefecture between January 2017 and November 2023. Clinical characteristics and follow-up outcomes were collected for analysis.

**Results:**

This study revealed 774 cases of brucellosis and 14 cases of BE, with an overall incidence rate of 1.88%. Most of the patients were male (71.43%) and lived in areas where brucellosis is common. Patients ranged in age from 26 to 68 years. Common symptoms reported among patients included chest tightness and fatigue, and a significant portion also presented with congestive heart failure. Most patients exhibited normal white blood cell counts (WBC) but had elevated levels of C-reactive protein (CRP). Transthoracic ultrasound (TTE) revealed cardiac valve vegetation in all patients, along with positive blood cultures. Six patients (42.86%) completed heart surgery, and ten (71.43%) completed anti-infection treatment. Six patients died, five of whom did not undergo surgery. The other patient with Marfan syndrome died after surgery. Sex, WBC count, neutrophil (NEUT) and total bilirubin (TBIL) were significant factors associated with regression in BE patients (*P* < 0.05) according to univariate analysis.

**Conclusions:**

Patients with BE in Kashi have a severe clinical presentation at diagnosis, but early detection with improved cardiac ultrasound and aggressive treatment can improve the prognosis.

## Introduction

Brucellosis is a zoonotic infection [5, 23, 24]. Brucellosis invading the heart mainly causes endocarditis, myocarditis, and pericarditis, among which Brucella endocarditis (BE) accounts for 1–2% [8], p. 590] and is the main cause of brucellosis-related death [5]. The clinical characteristics of Brucella-induced endocarditis are similar to those of endocarditis caused by other pathogens. More than half of the patients had symptoms of heart failure [3, 10], and even 80–90% were reported to have heart failure [17]. Chest tightness, shortness of breath, hepatosplenomegaly, enlargement of the heart boundary, and murmur in the lesion valve auditory area of the lesion are the main manifestations and signs [10, 11, 13]. A higher mortality rate was observed in patients who presented with typical clinical manifestations of cardiac insufficiency [16]. BE can occur in diseased valves, prosthetic heart valves, and normal valves [10]. The general data, epidemiological history, clinical features, laboratory tests and other medical records of 14 patients with BE were analyzed to provide a reference for clinical diagnosis and treatment.

## Data and Methods

### Research Subjects

This study examined all patients with BE admitted to The First People’s Hospital of Kashi Prefecture from January 2017 to November 2023. Brucellosis was diagnosed as Brucella infection by blood culture or vegetation culture. Transthoracic ultrasound (TTE) revealed glands with valvular redundancy, which was diagnosed as infective endocarditis.

The ethics committee of the First People's Hospital of Kashi approved this study with the code (2023) Expedited Review Study No. (89).

### Diagnostic Criteria

Brucellosis was diagnosed if any of the following criteria were met: (1) Brucella was isolated from blood, other body fluids, or tissue samples. (2) Patients who presented with typical clinical manifestations of brucellosis and a positive serum agglutination test (SAT) score ≥ 1:100.

Infectious endocarditis was diagnosed with reference to the modified Duke diagnostic criteria of 2023 [19].

### Inclusion and Exclusion Criteria

This study primarily focused on patients who had been diagnosed with BE. Patients with insufficient clinical data (< 80%) or lost to follow-up were excluded from the analysis.

### Clinical Data Collection

(1) Carefully ask the patient about the relevant epidemiologic history, including the raising of cows and goats and the presence of raw milk intake. (2) Clinical manifestations and signs, cardiac function, medical history, and other relevant information. (3) Laboratory tests included routine blood tests, procaicitonin (PCT), C-reactive protein (CRP), aminotransferases (ALT), albumin (ALB), coagulation function, blood culture and cardiac ultrasound results. (4) Treatment and prognosis.

### Statistical Methods

SPSS 26.0 was used for statistical processing. Descriptive methods were used to calculate the corresponding frequencies, composition ratios or occurrence rates. Normally distributed data are expressed as the mean ± standard deviation, and nonnormally distributed data are expressed as the median and interquartile range. Count data are expressed as cases (%). Univariate analysis was conducted using t tests for continuous variables and chi-square tests for categorical variables. *P* < 0.05 indicated that the difference was statistically significant.

## Results

### General Clinical and Epidemiological History

**Fig. 1:**
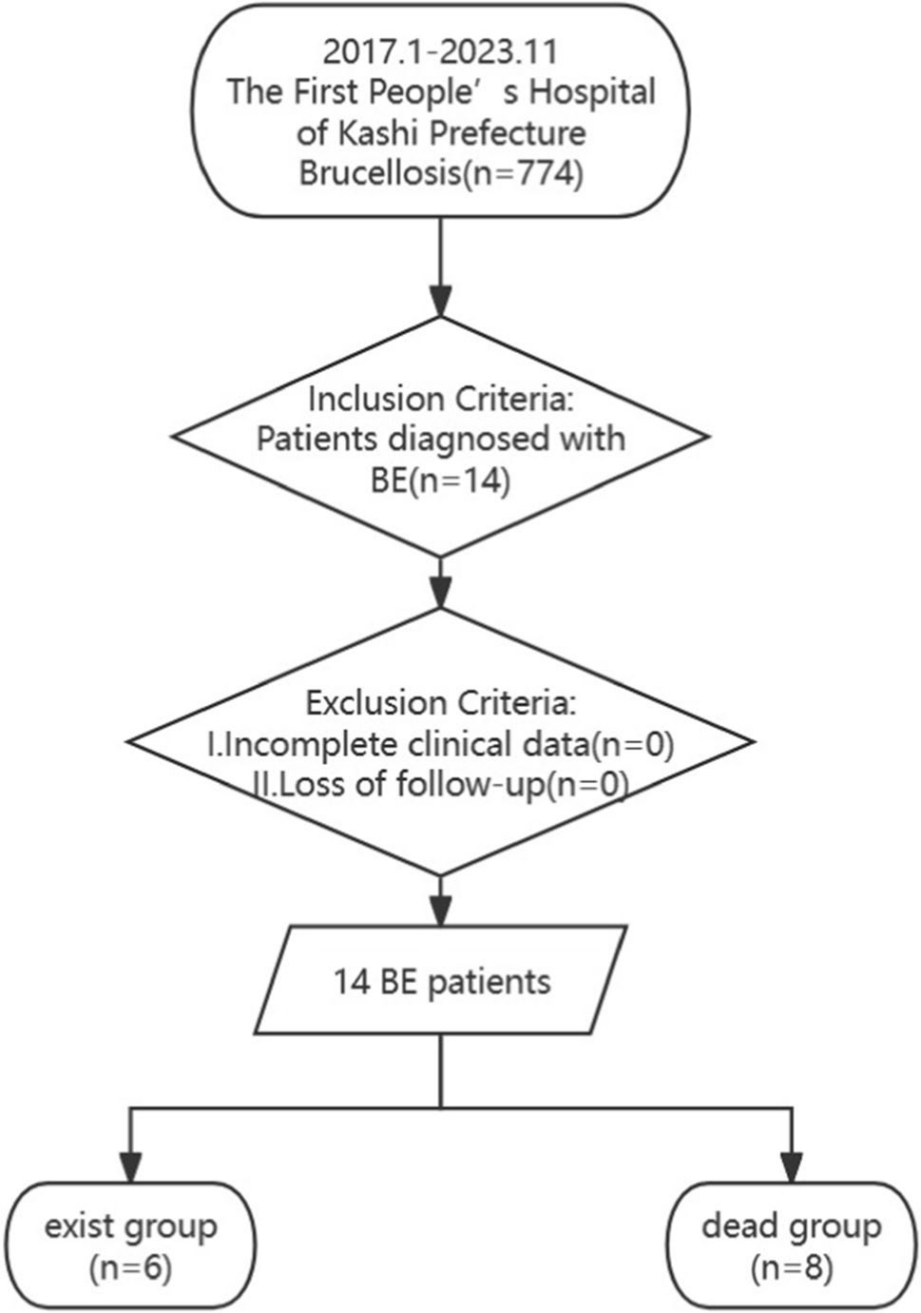
The flow of the cases screening

There were 774 patients with brucellosis and 14 patients with BE. All patients diagnosed with BE were monitored through telephone follow-up to ensure adequate clinical data be collected (Fig. 1). The incidence was 1.88%. The patients’ ages ranged from 26 to 68 years, with a median age of 49 years. All patients lived in areas where brucellosis is endemic, were Uyghurs, and had epidemiologic histories (see Table 1). Eight patients had basic heart disease. Four patients had rheumatic heart disease, one had Marfan syndrome, one had hyperthyroid heart disease, one had congenital heart disease, and one had coronary heart disease. Univariate analysis revealed that sex was associated with the time to death, with female patients being more likely to die. Age and underlying heart disease status were not significantly associated with time to death (Table 3).

**Table 1:** Clinical data, management, and outcome of 14 patients with Brucella endocarditis

Patient no.	1	2	3	4	5	6	7	8	9	10	11	12	13	14
Gender	Man	Woman	Man	Man	Woman	Woman	Man	Man	Man	Man	Woman	Man	Man	Man
Age (years)	37	26	60	38	41	51	68	35	54	56	53	38	47	54
Epidemiologic histories	Yes	Yes	Yes	Yes	Yes	Yes	Yes	Yes	Yes	Yes	Yes	Yes	Yes	Yes
Basic heart disease	No	No	No	Marfan syndrome	Hyperthyroid cardiopathy	Rheumatic heart disease	Congenital heart disease	Rheumatic heart disease	No	No	Rheumatic heart disease	No	Coronary heart disease	Rheumatic heart disease
Other underlying diseases	Cirrhosis	No	No	No	No	Cirrhosis	Stage 4 chronic kidney disease	Stage 4 chronic kidney disease	No	Stage 4 chronic kidney disease	Cirrhosis	No	No	No
Symptoms	Sweaty, weak, the mucous membrane yellow dye	Fever, sweaty, weak, stuffy, nausea and vomiting	Fever, sweaty, weak, stuffy	Fever, weak, stuffy, exertional dyspnea, orthopnea, nausea and vomiting	Weak, stuffy, exertional dyspnea, nausea, cough and sputum production	Fever, sweaty, bloating, abdominal pain and knee pain	Fever, weak, stuffy, exertional dyspnea, orthopnea, nausea , vomiting, cough and sputum production	Fever, weak, stuffy, exertional dyspnea, orthopnea, nausea , vomiting, cough and sputum production	Weak, exertional dyspnea and swollen feet	Weak, stuffy, exertional dyspnea and chest pain	Fever, weak, stuffy, exertional dyspnea, orthopnea, nausea , vomiting, cough, sputum production and oliguria	Weak, stuffy, exertional dyspnea, orthopnea, nausea and vomiting	Fever, stuffy and palpitation	Fever, sweaty, weak, , stuffy and palpitation
Signs	Negative	Negative	Negative	Systolic murmur	Systolic murmur	Diastolic murmur	Negative	Systolic murmur	Systolic murmur	Negative	Systolic murmur	Diastolic murmur	Negative	Diastolic murmur
Heart failure	No	Yes	No	Yes	Yes	No	Yes	Yes	Yes	Yes	Yes	No	Yes	Yes
Valvular regurgitation	Yes	No	No	Yes	Yes	Yes	Yes	Yes	Yes	No	No	Yes	Yes	Yes
Embolism	Spleen	No	Spleen	No	No	No	No	No	No	No	No	Spleen and kidney	No	No
Valve involved	Aortic	Mitral + tricuspid	Mitral	Mitral	Aortic	Mitral	Mitral	Mitral	Mitral	Aortic	Aortic	Aortic	Aortic	Aortic
Blood culture	Brucella melitensis	Brucella melitensis	Brucella melitensis	Brucella melitensis	Brucella melitensis	Brucella melitensis	Brucella melitensis	Brucella melitensis	Brucella melitensis	Brucella melitensis	Brucella melitensis	Brucella melitensis	Brucella melitensis	Brucella melitensis
Surgery	No	No	No	Yes	No	No	No	No	Yes	Yes	No	Yes	Yes	Yes
Anti-infective	No	Yes	Yes	Yes	No	No	Yes	No	Yes	Yes	Yes*	Yes	Yes	Yes
Outcome	Dead	Dead	Survived	Dead	Dead	Dead	Survived	Survived	Survived	Survived	Dead	Survived	Survived	Survived

*Despite anti-infective therapy, the patient’s condition continued to progress and she was discharged automatically. After her automatical discharge, adequate anti-infective therapy was not completed due to her progression. And she died at home 7 days later

### Clinical Performance

Most of the patients experienced chest tightness and fatigue, and more than half also experienced heart failure. Eight patients exhibited cardiac murmurs. The patient's clinical presentation was consistent with infective endocarditis (see Table 1).

### Auxiliary Examination

Most of the patients (11/14, 78.57%) had normal white blood cells, and eight (8/14, 57.14%) patients had elevated neutrophil (NEUT) percentages. All patients had CRP levels > 10 [60.0 (21.92, 73.89)] mg/L, and 8 patients had PCT levels > 0.25 ng/ml. Six patients experienced elevated transaminase levels, with the highest reaching 133.9 U/L. Hypoproteinaemia (ALB < 30 g/L) was observed in 9 patients, and the mean serum ALB concentration of all patients was 26.54 ± 6.18 g/L. The mean creatinine level for all patients was 83.0 (52.0, 139.2) µmol/L. Five patients had elevated creatinine levels (> 115 µmol/L), with the highest reaching 315.5 µmol/L. In combination with the medical history, three patients were considered to have acute damage, and two patients were considered to have chronic kidney damage. The aortic (7, 50%) and mitral (6, 42.86%) valves were the main valves affected, and 1 patient had combined mitral and tricuspid valves. The average left ventricular ejection fraction was 60.13 ± 7.64% (range 38.9–68%), which was < 50% of the two patients (14.29%). All patients (14/14, 100%) had positive blood cultures. Three patients had a spleen infarction, including one with double renal infarction (normal kidney function) (see Table 2).

**Table 2:** Auxiliary examinations of 14 patients with Brucella endocarditis

Patient no.	WBC*10^9^/L	NEUT, %	PLT*109/L	HBG, g/dl	PCT, ng/ml	CRP, mg/L	ESR, mm/h	ALT, U/L	ALB, g/L	TBIL, umol/L	DBIL, umol/L	Creatinine, umol/L	BNP	INR	D-dimer, μg/mL
1	4.22	80	47	8.5	3.01	65.17	12	35	19.7	75.2	50.6	185.4	53.3	1.36	6.93
2	8.85	82	21	8.2	0.246	101.01	85	133.9	28.5	29.1	0	83	775.7	1.35	4.47
3	3.04	45	23	10.2	0.062	60	2	99.4	18.7	22.8	0	40	1800	1.37	13.32
4	8.65	80	130	12.5	0.278	37.79	18	30	36.5	12	3	55	231.7	1.21	3.02
5	11.47	82	223	5.7	1.16	20.22	63	51	26.2	11.9	6.1	46.5	520	1.19	1.18
6	4.51	52	134	12.6	0.328	11.94	22	30	36.9	69.1	42.1	72	555.9	1.14	0.48
7	5.56	72	153	11.4	2.34	72.63	18	16	21.6	11.5	4	302.9	624.8	0.99	1.54
8	4.8	81	20	6.9	0.613	60.65	61	17	25.4	14	7.2	315.5	3874.44	4.28	5.93
9	5.83	65	269	11.8	0.24	23.61	37	16	34.6	6.5	2.3	60.5	16.9	1	0.66
10	4.92	67	84	6.5	1.02	75.15	47	42	21.7	5.1	2.4	221	589.1	1.23	2.83
11	10.28	88	138	7.4	0.975	104	87	8	19	38.6	26.5	84.2	526.8	1.73	4.81
12	4.16	75	65	11	0.7	53.2	28	106	26.6	49.9	4.3	84.8	854.3	1.39	7.53
13	8.03	61	266	10.7	0.078	16.19	9	27	30.5	19.7	9	52	NA	1.02	1.31
14	3.99	76	107	8.8	0.308	12.5	8	20	26.2	11.6	3.1	139.2	NA	1.13	1.13

WBC, white blood cell; NEUT, neutrophil; PLT, platelet; HBG, Hemoglobin; PCT, procalcitonin; CRP, C-reactive protein; ESR, erythrocyte sedimentation rate; ALT, alanine aminotransferase; ALB, albumin; TBIL, total bilirubin; DBIL, direct bilirubin; BNP, brain natriuretic peptide; INR, international normalized ratio

Reference range: WBC: 3.5–9.5; NEUT: 40–75; PLT: 125–350; HBG: 130–175; PCT: < 0.046; CRP: 0–4; ESR: 0–20; ALT: 5–40; ALB: 40–55; TBIL: 3.4–19; DBIL: 0–6.8; creatine: 62–115; INR: 0.95–1.50

### Treatment and Regression

All patients received active anti-infective and symptomatic and supportive treatment at admission but few of them dropped due to adverse drug reaction (ADR). 9 patients improved and 5 patients progressed when they discharge.

All patients were followed up by telephone, and the duration of follow-up ranged from 4 to 74 months, with a median follow-up time of 33.5 months. Six patients (42.86%) underwent cardiac valve surgery, while ten patients (71.43%) received antimicrobial therapy. A total of six fatalities (42.86%) were documented, with five occurring among patients who did not successfully undergo the surgical procedure. In the eleventh case, the patient's condition progressed to ventricular fibrillation during hospitalization, ultimately resulting in the patient's death 7 days after discharge. In Case 4, the patient was discharged from the hospital with anti-infective treatment for 2 months. Two months later, relevant valve replacement surgery was performed. After surgery, the surgeon considered that rifampicin would affect the effect of anticoagulant drugs and advised the patient to monitor coagulation during medication. However, the patient discontinued the drug spontaneously after discharge and died at home 6 months later, and the specific cause of death was not clear. Four patients died at home after being discharged from the hospital, and the cause of death is unknown.

### Univariate Analysis of Factors Affecting the Regression of BE Patients

Univariate analysis revealed that sex, white blood cell count, absolute neutrophil count, globulin and total bilirubin were all influential factors in the regression of patients with BE (*P* < 0.05) (Table 3).

**Table 3:** Univariate analysis of factors affecting the regression of BE patients

Factor	Exist group (n = 8)		Dead group (n = 6)		*P*
Age (year)					0.58
<40	2	25	3	50	
≥40	6	75	3	50	
Gender					*0.02*
Man	8	100	2	33.33	
Women	0	0	4	66.67	
Fever					0.59
Yes	5	62.5	2	33.33	
No	3	37.5	4	66.67	
HBG (g/L)					0.59
<90	3	37.5	4	66.67	
≥90	5	62.5	2	33.33	
Kidney injury					
Yes	4	50	1	16.67	
No	4	50	5		
Valve involved					0.46
Mitral	4	50	2	33.33	
Mitral + tricuspid	0	0	1	16.67	
Aortic	4	50	3	50	
Underlying heart disease					0.59
Yes	2	25	4	66.67	
No	6	75	2	33.33	
Cirrhosis	0	0	3	50	0.055
Chronic kidney disease	3	37.5	0	0	0.209
Heart failure					1
Yes	6	75	4	66.67	
No	2	25	2	33.33	
Size of vegetable (mm)					0.59
<15	3	37.5	2	33.33	
≥15	5	62.5	4	66.67	
Embolism	2	25	1	16.67	1
Surgery					0.14
Yes	5	62.5	1	16.67	
No	3	37.5	5	83.33	
Anti-infective					0.25
Yes	7	87.5	2	33.33	
No	1	12.5	4	66.67	
WBC (×10^9^/L)	5.04±1.50		8.00±3.00		0.03
NEUT (×10^9^/L)	3.43±1.03		6.39±2.91		0.02
PLT (×109/L)	95.5 (34.0, 237.75)		134 (34.0, 180.5)		0.84
PCT, ng/ml	0.31 (0.08, 1.02)		0.65 (0.27, 1.62)		0.53
CRP, mg/L	60.0 (23.61, 72.63)		51.48 (18.15, 101.76)		0.78
ESR, mm/h	28.0 (8.0, 47.0)		22.0 (15.0, 74.0)		
ALT, U/L	23.5 (16.25, 85.05)		35.0 (19.0, 92.45)		0.73
ALB, g/L	25.66±5.14		26.06±7.31		0.91
TBIL, umol/L	12.8 (3.05, 22.03)		38.6 (20.5, 72.15)		0.04
Creatinine, umol/L	84.80 (52.0, 221.0)		71.43±17.50		0.30
BNP	739.55 (446.05, 2318.61)		523.40 (187.10, 610.85)		0.17

WBC, white blood cell; NEUT, neutrophil; PLT, platelet; HBG, Hemoglobin; PCT, procalcitonin; CRP, C-reactive protein; ESR, erythrocyte sedimentation rate; ALT, alanine aminotransferase; ALB, albumin; TBIL, total bilirubin; BNP, brain natriuretic peptide; INR, international normalized ratio

## Discussion

BE is primarily documented through case reports or retrospective analyses, with an incidence rate of 1–2% [8]. The First People's Hospital in Kashi has admitted 774 patients with brucellosis in the last 6 years. Fourteen patients had BE. The incidence rate was 1.88%. Most of them were males involved in animal husbandry, which is largely consistent with the existing prevalence of brucellosis [14].

In this study, the most common manifestations were fatigue, chest tightness, and dyspnea during exertion, with eight patients experiencing congestive heart failure. The fatality rate was 42.86%, which is in general in agreement with the high case fatality rate of cases in the available literature [22]. The clinical presentations of the 14 patients in this study were generally similar to those of patients with BE caused by other pathogens and could only be further differentiated by pathogenetic findings.

The heart valves invaded in this study were mainly aortic and mitral valves, including a case of a mitral valve prosthesis. The findings were in line with the valve involvement described in the literature [15]. The majority of patients presented with solitary vegetation, with more than half of the valve vegetation measuring > 10 mm in diameter. All valve vegetation in the patients was detected via TTE. In this study, three patients (21.43%) presented with concurrent splenic infarction. The risk of embolism is very high in patients with infection endocarditis (IE), with embolic events occurring in 20–50% of patients. The brain and spleen are the most frequent sites of embolism in left-sided IE. Embolic events are frequent and life-threatening complications of IE related to the migration of cardiac vegetation, and large (≥ 10 mm) vegetation has a greater incidence of embolism [7].

Most reported cases of BE are diagnosed based on positive blood cultures and/or positive serum agglutination tests [3, 12, 21]. Although a positive blood culture is the gold standard for diagnosing IE, the rate of positivity can vary widely [4]. All patients in this study had positive blood cultures, likely because most of them showed symptoms of congestive heart failure, such as chest tightness and shortness of breath. Only 28% of the patients had fever symptoms and did not receive multiple antibiotic treatments for infection.

*Brucella melitensis* was identified in all patients included in this study. This observation may be attributed to the predominant etiological role of *Brucella melitensis *in cases of brucellosis in Xinjiang, China [6], as well as the higher severity of illness among the study participants, which is commonly associated with *Brucella melitensis* infection.

It is difficult to diagnose Brucella endocarditis early on. When the 14 patients in this study were diagnosed, 11 had valve-related regurgitation, and 10 had heart failure. Due to late diagnosis, most patients had localized heart damage and a poor prognosis, with a mortality rate of 42.86%.

Without effective anti-infection and heart valve replacement treatment, the mortality rate is almost 80%. Patients who received effective anti-infec valve replacement surgery had a good prognosis [9, 18, 20]. In this study, 6 patients underwent valve replacement surgery and anti-infection treatment. Among the 8 patients who did not undergo surgery, 5 passed away. One patient died despite surgery, likely due to Marfan syndrome. The shortest survival period was 7 days, and the longest was 27 months. For patients with BE, effective anti-infection treatment combined with valve surgery significantly impacts survival.

The results of the univariate analysis indicated that individuals who were female, presented with high white blood cell counts, and had elevated bilirubin levels were at a higher risk of mortality. Our study found a higher mortality rate in women with BE, but more research with larger sample sizes is needed to understand the underlying mechanism. Elevated bilirubin levels have been implicated in increased mortality rates among patients, especially those with concurrent cirrhosis. Although univariate analysis did not show statistics significant impact of cirrhosis on patient outcome, we found that all patients with cirrhosis died. We further analyzed the conditions of all patients before discharge. The first patient, treated with rifampicin, doxycycline, and levofloxacin, developed drug-induced liver and kidney function impairment and requested automatic discharge. The sixth patient, treated with doxycycline and ceftriaxone, was switched to sulfamethoxazole and trimethoprim (SMZ-TMP) and gentamicin after disease progression. However, the patient developed drug-induced renal impairment and requested automatic discharge. In the eleventh patient, rifampicin and doxycycline were administered, however the patient developed recurrent ventricular fibrillation and requested automatic discharge. Although all patients with cirrhosis have died, not all of their deaths were related with liver conditions. Patients with cirrhosis are in a state of relative immunosuppression for a long time [2] and are also more likely to develop drug-related liver damage than healthy individuals [1]. However, whether cirrhosis is an important factor contributing to the occurrence of adverse outcomes in patients with BE may require further study with an expanded sample size.

This study is the largest clinical study on BE conducted in China, with the largest sample size to date. The patients included in the study were from the Kashi region of Xinjiang, China, an area where tuberculosis is common. This suggests the possibility of concurrent tuberculosis. The medications used to treat both brucellosis and tuberculosis may reduce the probability of detecting positive brucellosis cultures. Therefore, in regions with a high prevalence of brucellosis, if standard anti-infective therapy is not effective for IE, healthcare providers should consider the possibility of concurrent brucellosis and use brucellosis serologic testing as an additional diagnostic tool.

The main limitation of this study is the small number of patients included. In the future, we plan to work with multiple brucellosis clinics to analyze the medical records of more patients with brucellosis endocarditis. Additionally, due to limited access to quality medical care in Kashi, diagnoses are often delayed, resulting in late-stage disease identification. While improving the standard of diagnosis is the best solution, it will take a long time to make these changes.

## Conclusion

Brucella endocarditis is rare in the Kashi region and usually affects the aorta and mitral valve, often causing heart failure. Diagnosis is usually delayed, leading to patients showing localized heart damage and having poor prognoses. Improving cardiac ultrasound exams can help detect Brucellosis endocarditis earlier, and regular anti-infection treatment combined with valve replacement surgery is an effective treatment approach.

## Data Availability

The original contributions presented in the study are included in the article. Further inquiries can be directed to the corresponding authors.

## Declarations

### Generative AI and AI-Assisted Technologies in the Writing Process

During the preparation of this work, the author (s) utilized chatGPT for critical revision of the manuscript. Subsequently, the author (s) reviewed and edited the content as necessary and assumed full responsibility for the publication's content.
